# Beyond Residents-as-Teachers: The Development of an Advanced Medical Education Pilot Elective for Pediatric Residents

**DOI:** 10.7759/cureus.40937

**Published:** 2023-06-25

**Authors:** Christina M McKinney, Rebecca Hart, Adam C Patterson

**Affiliations:** 1 Pediatrics, University of Louisville School of Medicine, Louisville, USA; 2 Pediatric Emergency Medicine, University of Louisville School of Medicine, Louisville, USA

**Keywords:** advanced medical education, medical education curriculum, residency curriculum, medical education, resident education, curriculum development, medical education elective, kerns six steps

## Abstract

Introduction

An advanced medical education elective can encompass themes that transcend traditional residents-as-teachers curricula. The literature is scarce regarding the development of such a curriculum for pediatric residents.

Objectives

To develop and implement an advanced medical education elective for pediatric residents and evaluate the effectiveness of the educational strategies and curriculum.

Methods

Kern’s Six Steps were applied to create a two-week-long elective for pediatric residents. Residents worked through Kern's model to collaboratively develop the elective. Faculty were recruited based on expertise and content previously created. Residents developed teaching sessions for fourth-year medical students and received feedback. The curriculum was evaluated using quantitative and qualitative feedback with a five-point Likert scale and open-ended questions, group discussions, elective evaluations, and the New World Kirkpatrick Model.

Results

Five residents, 17 students, and 22 faculty participated. Lectures, expert panels, group discussions, and teaching sessions were seen as effective instructional methods. All residents were satisfied with the elective and its strategies and developed useful skills. Resident-led teaching sessions and interactive learning strategies were cited as a strength, while some redundancy was noted as a weakness. Faculty recommended more formal feedback on resident-led teaching sessions in the future.

Conclusions

Our medical education elective was designed collaboratively with residents on a medical education track. Strong faculty participation, asynchronous learning, and resident-led teaching sessions were strengths of the curriculum. The curriculum’s reproducible components may serve as a foundation for institutions interested in improving their medical education didactics for residents. More research is needed to determine the external validity of this novel curriculum.

## Introduction

Training residents to become medical educators is an invaluable skill that has implications on their abilities to educate learners and promote understanding among healthcare teams. However, the concept of “teaching” does not exist on its own as a core competency in resident education [1]. Medical students receive approximately one-third of their teaching from residents [2]. However, despite the important role that residents play in medical student education, there is no common conceptual framework that outlines the skills required for a resident to become a medical educator [3].

To improve residents’ skills in this area, residents-as-teachers curricula have become increasingly adopted by graduate medical education programs [4,5]. However, such programs are often generalized and limited in scope, focusing on bedside or didactic teaching for residents whose primary goal is outside the educational sphere. By contrast, medical educators at some point “make a conscious transition into a new identity,” requiring ongoing development of more advanced skills [6]. More comprehensive medical education curricula can encompass themes that transcend the traditional residents-as-teachers curriculum and emphasize higher-level goals, including professional identity formation, educational scholarship, networking, and curriculum design. An advanced and comprehensive medical education curriculum can provide a holistic approach for residents interested in careers in medical education, but the literature is scarce regarding the development and implementation of such a curriculum in residency.

To fill this gap in medical education didactics, we sought to develop an advanced medical education elective for pediatric residents using a framework that would be easily adaptable for use at other institutions as well as our own. Our aims included the following: (1) to evaluate each educational strategy’s effectiveness in fulfilling the elective goals and objectives, (2) to assess residents’ perceived competence as educators prior to and following participation in the elective, and (3) to systematically evaluate the effectiveness of the elective and its reproducibility at other institutions.

## Materials and methods

**Figure 1:**
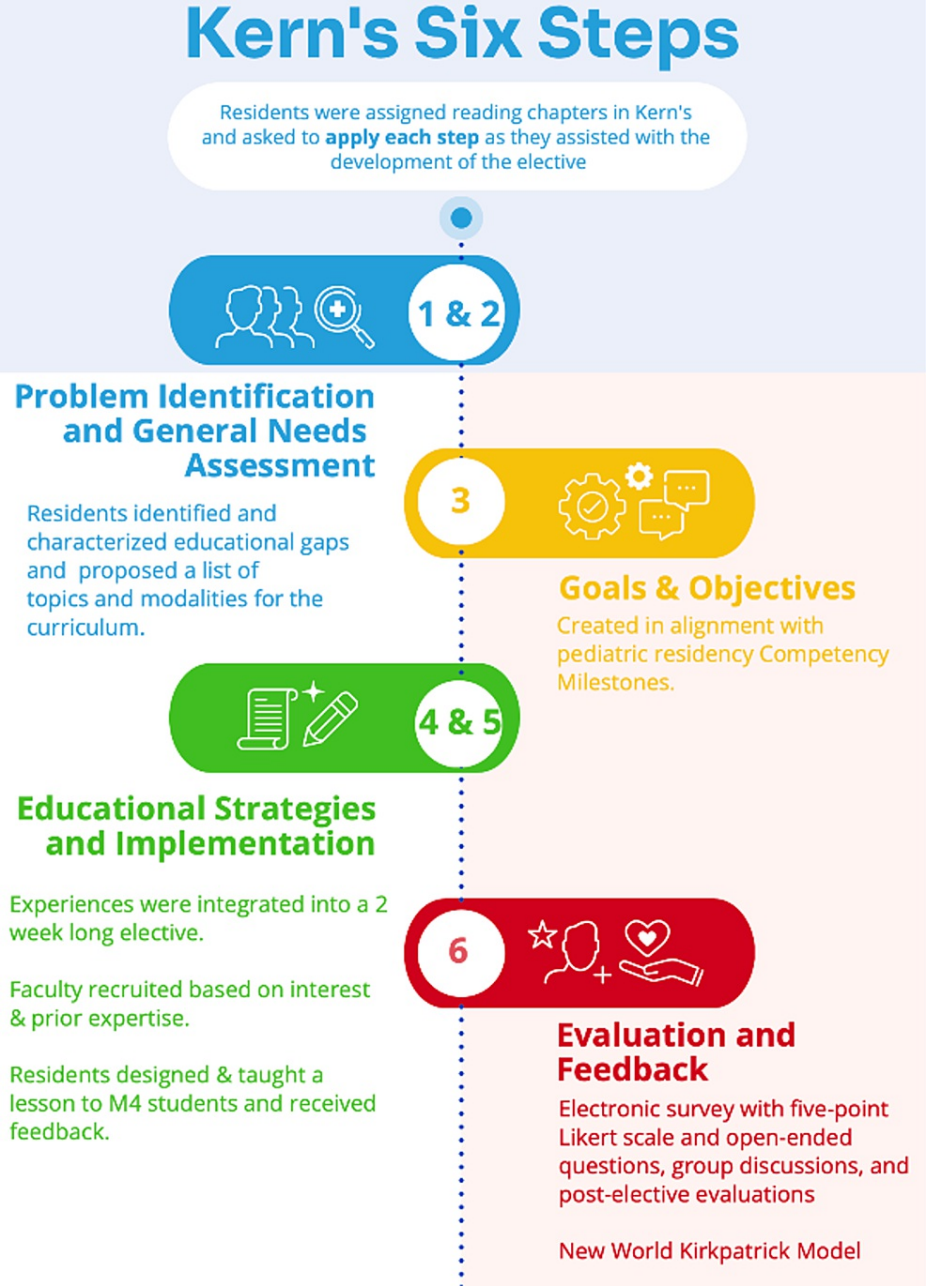
Six-Step Approach to the Development of an Advanced Medical Education Elective

A medical education distinction track [7] was developed at our institution (a tertiary care, freestanding pediatric facility with approximately 24 categorical pediatric residents, five combined internal medicine-pediatrics, and three child neurology residents per year) in 2019 and is available to any interested resident starting in the second half of intern year. As part of the distinction track, the advanced medical education elective was subsequently developed at the beginning of the academic year in 2021 with the active input and assistance of track participants. Utilizing asynchronous learning, residents were assigned reading chapters in Curriculum Development for Medical Education: A Six-Step Approach [8] and then asked to apply each step as they assisted with the development of the elective (Figure 1). 

Problem identification and targeted needs assessment (Steps 1 and 2)

Residents identified and characterized gaps in the topic of medical education that they had noticed during their training in medical school and residency and proposed a list of topics to include in the curriculum as well as suggestions for modalities to achieve these goals.

Goals and objectives (Step 3)

The problem identification and needs assessments from the prior steps informed the development of the elective goals and objectives in alignment with pediatric residency Competency Milestones [1]. These goals and objectives underwent cognitive review with our institution’s Program Evaluation Committee prior to finalization.

Educational strategies and implementation (Steps 4 and 5)

The needs assessment and evidence-based goals and objectives were utilized to develop a variety of educational experiences. These experiences were integrated into a two-week-long medical education elective. The structure and optimal timing of experiences and didactics within the schedule were determined based on participating resident and faculty availability. Faculty members from pediatrics and medicine-pediatrics were recruited based on expertise in medical education and were invited to utilize educational materials that they had previously created and presented, in alignment with the stated needs and goals/objectives of the course. We collaborated with “Practical Pediatrics,” a transition to residency course for fourth-year medical students applying to pediatrics at our institution, to provide opportunities for hands-on teaching sessions. Residents were given dedicated time during the elective to create learning objectives and design and teach a lesson plan of their choice for Practical Pediatrics students. Residents provided each other with verbal feedback both prior to and following their teaching sessions. Medical students also provided the residents anonymous written with feedback following their teaching sessions, which will be used to improve the sessions for next year.

Evaluation and feedback (Step 6)

The curricula were evaluated using both quantitative and qualitative feedback from faculty, residents, and medical students, utilizing individual session evaluations, group discussions, and post-elective participant evaluation regarding satisfaction, medical education skill development, and professional identity formation. Data were collected via an electronic survey (Qualtrics XM), as well as via semi-structured group discussions of participants. Resident satisfaction, perceived skill development, and the effectiveness of instructional methods in achieving the goals were evaluated utilizing Likert-scale questions on a range of 1 (strongly disagree) to 5 (strongly agree). 

Following completion of the course, an electronic survey was distributed to residents who participated in the elective with open-ended questions. Questions revolved around new skills acquired, positive outcomes of the elective, and improvements that could be made for future iterations of the course. Medical student perceptions of the helpfulness of the resident-led teaching sessions were evaluated with Likert scale questions on a range of 1 (very helpful) to 5 (very unhelpful). Due to the limited number of participants available for this pilot study, a more detailed qualitative methodology with inductive or deductive coding was not utilized, but representative quotes of common themes were identified by investigators and reported.

A framework proposed by Li et al. that focuses on the New World Kirkpatrick Model (NWKM) was utilized to holistically evaluate the curriculum in order to examine what was effective and how and why the elective contributed to the outcomes observed [9,10].

## Results

**Figure 2:**
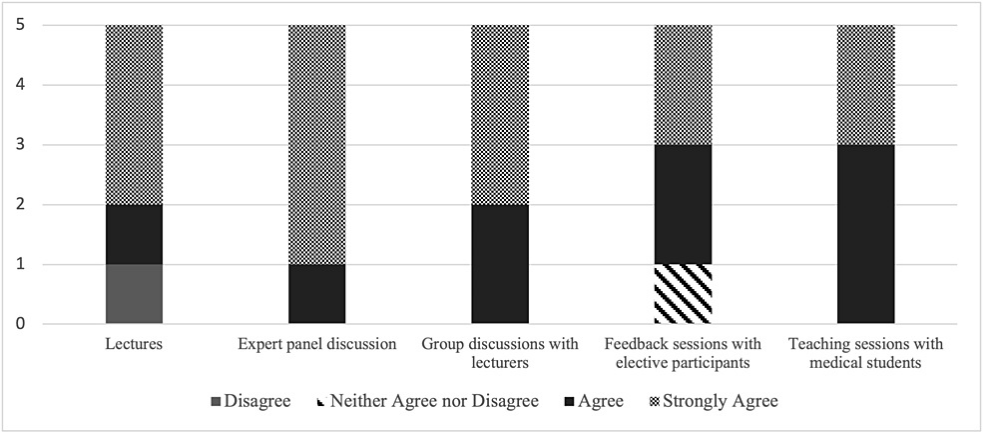
Effectiveness of instructional methods in achieving goals and objectives.

**Figure 3:**
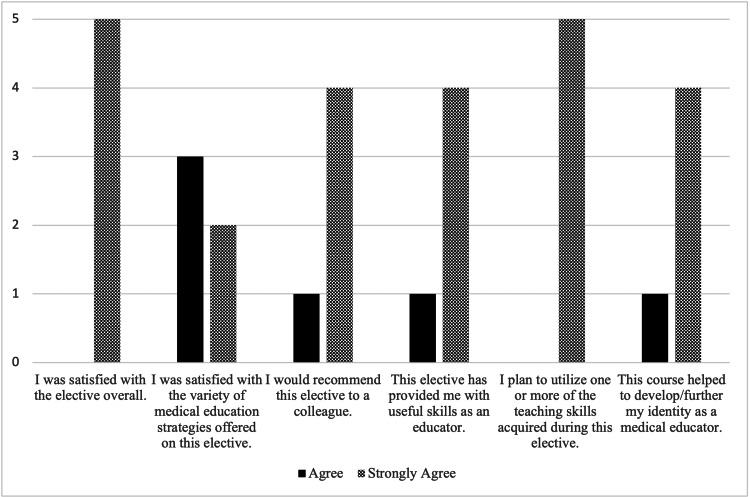
Post-curriculum resident satisfaction and perceived skill development.

Five residents (three pediatrics, two medicine-pediatrics), 17 medical students, and 22 pediatrics and medicine-pediatrics faculty presenters participated in the pilot elective course. Lectures, expert panels, group discussions, feedback sessions, and teaching sessions were seen as effective instructional methods (Figure 2). All residents were satisfied with the elective and its educational strategies, developed useful skills as a medical educator and plan to utilize skills acquired during the elective, and believed that the elective helped develop their identities as medical educators (Figure 3). All residents would recommend the elective to a colleague (Figure 3). Most medical students found the resident-led sessions to be “very helpful” or “somewhat helpful,” which included simulated parent phone calls (82%, 14/17), a procedure skills workshop (94%, 16/17), patient handoffs (94%, 16/17), a “sick or not sick” session (94%, 16/17), and a neonatal simulation session (88%, 15/17). 

Responses to open-ended questions from semi-structured online feedback sessions for residents and faculty are listed in Table 1 and Table 2, respectively. 

**Table 1:** Qualitative Feedback From Resident Participants

Question	Resident Response
Which skills are you most likely to incorporate into your practice as a medical educator?	“Interactive methods for group learning to encourage knowledge retention and critical thinking.”
	“How better to teach and use graduated learning.”
	“Using the 5 min preceptor method for teaching during rounds.”
	“Composing goal and aim statements for lectures or research.”
	“How to develop curriculum and learning objectives.”
	“How to incorporate technology into teaching.”
What were the strengths of the elective?	“Incorporation with the Practical Pediatrics group was incredible. I really loved the ability to work directly with [4^th^ year medical students] and put what we were learning into action!”
	“Inviting a wide array of faculty speakers with their own interests and passions for Med Ed was also very helpful in hearing their perspectives and journeys.”
	“Discussing the theory of adult learning and different methods for interactive learning in groups.” “The practical time to teach medical students, critical thinking and teaching sessions, access to and discussion with med ed leaders.”
What were the weaknesses of the elective?	“Some sessions were repetitive.”
	“Would like more free time to advance [scholarly] projects.”

**Table 2:** Qualitative Feedback From Faculty Participants

What is your role in medical education in our department?	What was the topic of your session?	What were the objectives of your talk?	What is the ideal target audience for your talk?	My session reached the goals and objectives for the elective.	What would you change for the next rotation?
Director of Research Development	Lifelong learning across the continuum	Review the importance of “fit” in careers in medical education through the lens of a career path; Discuss the utility of “why” and collaboration in medical education scholarship; Consider opportunities for career development in medical education in 2022; Review an example of curriculum development with the goal of developing lifelong learning skills	Upper level residents, Fellows	Agree	I can see where it might be useful to the learners to actively teach something and get expert feedback.
Associate Program Director, Director of Scholarly Activity	Journal Club and Med Ed Scholarly Project Presentations (SOC)	Review common methodologies for evaluation of medical education- based literature and practice the use of those tools; Discuss current progress of residents' scholarly activity and review pearls and pitfalls for completion	Upper level residents	Strongly agree	…maybe it would be better to have the articles that residents plan to present for journal club ahead of time, where people could review and develop comments that could generate additional discussion.
Pediatrics Residency Advocacy Director	Op-ed writing session	Describe op-eds and who writes them; Outline an approach to writing an op-ed; Discuss ideas for an op-ed about food insecurity	Medical students, Interns, Upper level residents, Fellows	Agree	I think this would have benefited from having a clearer topic chosen in advance so we would be able to practice writing.
Pediatrics Residency Advocacy Director	Mentorship	Define mentor, coach and sponsor; Explore personal experiences of the group as a mentee and a mentor; Reflect upon our own mentoring needs; Discuss tips for successful mentor-mentee relationships	Medical students, Interns, Upper level residents, Fellows	Agree	I could have done better facilitating conversation from students about previous experiences by asking more open-ended questions.
Co-Course Director, Practical Pediatrics	Reviewing Curriculum Development through the Lens of Practical Pediatrics	Discuss the evolution of Practical Pediatrics and the incorporation of more hands on learning experiences	Interns, Upper level residents	Agree	[I] may want to incorporate more evidence- based facts about effective learning methods in relation to Practical Pediatrics.
Residency Program Director	Teaching Clinical Reasoning	Walk through process of clinical reasoning in the setting of a patient; Identify different elements of clinical reasoning; How problems with certain elements might present; Strategies for helping students or more junior residents develop their clinical reasoning skills	Interns, Upper level residents, Fellows, Faculty	Agree	…with more time, I could make it more of a workshop with learners in difficulty.
Former Program Director, Current Department Chair	Careers in Medical Education	Panel discussion with the residents about pathways for careers in med-ed and how to navigate these and what might be needed	Upper level residents, Fellows, Faculty	Strongly agree	Consider a little more time and some pre-asked questions so we can guide our story-telling in the right direction.
Chief Resident	Feedback in Transitional Roles	Help the group identify what defines quality feedback vs examples of less helpful feedback; Discuss effective ways to give feedback; Explore how to seek and incorporate feedback.	Interns, Upper level residents	Agree	[I] would consider including students in the conversation if able moving forward since they represent another important component of the team.
Associate Program Director, Facilitator of Humanism and Compassion in Medicine/Problem Based Learning, MS2 Disease & Therapeutics Thread Director for Female Repro & Liver/GI, Clinical Skills Committee, Evaluation & Assessment Subgroup, Student Promotions Committee	Medical Education Innovations	Lessons through my journey: Keep your eyes open, make connections and ask if things can be done differently, raise your hand for something new, look at other disciplines, take advantage of free training, embrace your skills & reputation to mentor others	Medical students, Interns, Upper level residents	Strongly agree	I talked a lot about my journey and it really seemed this group wanted to talk more about [undergraduate medical education] as they hadn't had as much on this.
Course Director, Practical Pediatrics	Several sessions embedded in Practical Pediatrics this year	To increase students' comfort with the following intern functions: ER call ups/handoffs, Parent pager calls, Rounds presentations, Teaching during rounds	Medical students	Strongly agree	I think that the intersection of the Practical Pediatrics elective with the Resident Med Ed elective is a perfect pairing- we will have a new audience of 4th year medical students going into Peds every year, which represents what I would consider an ideal audience for Residents' Med Ed educational sessions.

## Discussion

This novel medical education elective for pediatric residents was designed collaboratively with residents on a medical education distinction track, with high levels of satisfaction, improvement in perceived competence as medical educators, and development of skills and professional identity as notable achievements after evaluation of the pilot course. Kern’s Six-Step Approach to Curriculum Development is frequently utilized in pediatric resident education and has been shown to improve resident satisfaction and knowledge in several domains [11-14]. While it has been used to develop several curricula in resident education, literature is scarce regarding its specific use in the implementation of a medical education elective for pediatric residents [11-14].

Utilizing a framework proposed by Li et al. in conjunction with the NWKM, we examined the strengths of our curriculum and opportunities for improvement in coming years by examining the four levels of learning identified by Kirkpatrick, including reaction, learning, behavior, and results of a curriculum or educational intervention [9,10].

Level 1: reaction

We obtained participant satisfaction data that suggested that our educational strategies were favorably received, the elective fulfilled its goals and objectives, and the experience contributed to a deeper understanding of medical education. 

Level 2: learning

All residents noted that the elective provided them with useful skills as an educator, that they plan to utilize one or more of the teaching skills acquired during the elective, and that the course helped develop/further their identities as medical educators. 

Level 3: behavior

The elective emphasized asynchronous learning, giving residents the opportunity to read and apply their knowledge by working through the steps of Kern's model to collaboratively develop the elective, rather than merely participate in it. Importantly, residents were given dedicated time during the elective to create resident-led sessions, encouraging skill acquisition with real-time feedback from faculty and peers. We emphasized the resident-led sessions as a core component of the educational experience, as resident-led teaching has been shown to enhance student education and satisfaction and leads to improved job satisfaction and knowledge acquisition for residents [15-19]. This cohort of residents on the medical education track will lead these sessions again in subsequent years, so they will be able to incorporate feedback to improve their sessions. Ongoing assessments in the future may include following the identified residency competency milestones and/or teaching evaluations of participants over the course of their training and beyond to evaluate the integration of these skills into practice as educators [19,20].

Level 4: results

Our elective was an extension of a medical education track in the program, which included an audience of learners who had a distinct interest in advanced medical education didactics. Embedding this elective into the framework of a medical education track enabled us to simultaneously fulfill the larger goals of the residency program by creating advanced didactics for medical educators that aligned with the requirements of the medical education track. Furthermore, allowing a full two-week curriculum created a unique opportunity for residents to network with learners and experts in the field and build a community of medical educators, which is an important component of professional identity formation [6].

As this was a pilot group, this study was limited by the small sample size of participants at a single site. While the site and implementation strategies used here were favorably received by participants at our institution, they may not be generalizable to all programs depending on resident, faculty, and student availability. However, we believe that the principles and strategies demonstrated may be adapted by other residency programs interested in developing formal, advanced medical educational didactics. Specifically, the use of Kern's Six-Step Process demonstrates adaptability and ability to engage interested residents in the elective development process, allowing individual institutions to identify their own needs and develop goals and objectives to meet them where they differ from our own. Programs that do not have a “transition to residency” cohort or medical student boot camp may, for example, consider focusing such a curriculum toward rotating third-year or sub-intern medical students on their pediatrics rotation or other interested learners who are planning to apply to pediatrics.

The schedule was created to ensure that we did not overburden the faculty. Faculty were recruited based on medical education expertise, so most, if not all, faculty were able to use prior learning materials that they had previously developed as opposed to having to design new lesson plans. Other institutions can similarly adapt this technique for scheduling by drawing upon preexisting material and designing a curriculum around the strengths within their respective departments to enhance faculty participation. 

Curriculum design is an iterative process that focuses on assessing and improving over time. While this pilot program demonstrated success and feasibility, we plan to continue to improve and adjust this curriculum in the coming years. While we have provided a starting point for other institutions to customize our process to improve the medical education didactics available for their learners, more research is needed to determine the external validity of this novel curriculum.

## Conclusions

We created a medical education elective that improved resident satisfaction with medical education didactics within our program and enhanced residents’ perceived competence as medical educators and enhanced professional identity formation. Strengths of the elective included a combination of strong faculty participation, asynchronous learning, and emphasis on practical skill application using resident-led teaching sessions. While the curriculum’s reproducible components may serve as a foundation for other institutions interested in improving their medical education curricula for residents, more research is needed to determine the external validity of the curriculum.

## Appendices

**Table 3:** Targeted Needs Assessment

Question	Resident Response
Identify and characterize the health care problem that will be addressed by the curriculum, how the problem is currently being addressed, and how it ideally should be addressed.	Many residents are interested in participating in medical education during their professional career. Currently, formal curriculum on adult learning and education varies widely among residency programs.
	The University of Louisville pediatrics residency does not have a formal medical education elective to address the many ways physicians may be asked to educate others throughout their careers.
	The dedicated med ed elective will teach residents the foundations of medical education, including how to create new curriculum and further develop specific med ed associated research projects pertaining to their interests. Through readings, presentations and guided discussions, residents will become more proficient in the basics of medical education.
	The medical education track will help residents perform a general needs assessment for their own projects to improve their skills in medical education.
What are the educational gaps in the topic of medical education that you have noticed thus far in your training?	Lack of formal structured education for resident to learn principles of adult learning
	Need for improved education about efficient bedside teaching
	Minimal instruction about how to teach medical students
	Lack of education about curriculum development
	Limited opportunities for residents to learn about or participate in med ed basics
	Lack of knowledge regarding appropriate resources to use for learning about medical education
Make a list of at least 3 topics that would be helpful to include in our medical education curriculum.	Adult learning theory
	Bedside teaching/teaching during rounds
	Curriculum development
	Structuring questions at the level of the learner
	Methods of developing an efficient/engaging lecture
What is the best way to complete these didactics?	In person sessions Online modules
	Reading assignments
	Group sessions for project feedback
	Practice bedside teaching
	Journal club
	Hands on experiences
Are there any specific experiences that you would like to complete?	Opportunity to teach medical students
	Lead simulations and/or structured physical exam and clinical teaching
	Develop a teaching session for medical students using basics of curriculum development
	Writing our own goals and objectives

**Table 4:** Elective Goals and Objectives

Competency Milestone	Goal		Objectives/Activities
Patient Care		To explore how medical education principles translate to improved patient care	The resident will: Be introduced to simulation in medical education, explore principles of crisis resource management and practice implementation of case scenarios. Explore evidence-based techniques and educational strategies in history taking/completing physical exams, communication, clinical reasoning and consider their effects on patient outcomes.
Medical Knowledge		To explore, understand and apply medical education principles and topics (e.g. adult learning theory, feedback and evaluation, mentoring, coaching, curriculum development)	The resident will: Develop an in-depth understanding through scheduled didactics, modules, expert discussion, self-directed study. Implement this learning in educational sessions that are developed for 4^th^ year medical students and track participants, with associated reflection, feedback, and revision.
		To improve understanding of evidence-based literature and the process of scholarly activity in the field of medical education.	The resident will: · Be introduced to the principles and design of medical education and qualitative research. Participate in medical education journal club. Present current progress on their scholarly activity, with focus on literature review, research methods, and outcomes, and use protected time to advance their project.
Systems Based Practice		To introduce basic principles of patient safety and quality improvement and explore their connection to medical education.	The resident will: Review principles of patient safety and quality improvement. Explore best practices in patient safety and quality improvement curricula and implementation practices.
Practice Based Learning and Improvement		To improve understanding of the breadth of scholarly activities and academic expectations for academic faculty with focus on teaching excellence.	The resident will: Perform a career-focused self-assessment and update his/her CV. Meet with faculty and/or fellow mentors for CV review and discussion about next steps during residency (e.g. mock interview, preparation for fellowship, completion or initiation of scholarly activity).
Professionalism		To demonstrate professional behavior in all dealings with colleagues, students, and preceptors.	The resident will: Conduct interactions with a professional mindset, sense of duty, and accountability. Attend and participate in scheduled sessions. Complete rotation evaluation and obtain feedback from faculty during rotation.

**Table 5:** Pilot Medical Education Curriculum, February 2022

Monday	Tuesday	Wednesday	Thursday	Friday	
8 -11 AM - Practical Peds (PP) project preparation	8:30 AM - Morning report	8:30 AM - Morning report	8:30 AM - Morning report	8 AM - Grand rounds	
	9 AM - PP project preparation	9 AM - "Sick or Not Sick" for PP	9 AM - Journal club	9 AM – Question Aid for Rich, Realtime Discussion Workshop (Faculty K)	
	10:30 AM - "Teaching Mastery of Procedures" (Faculty F)	10:30 AM - Resident Teaching Session - "Handoffs"	10 AM - Brief Scholarly Project Updates	10 AM - Bloom's Taxonomy (Faculty K)	
11 AM - "Securing your Future" (Faculty A)			11 AM - "Scholarly Frameworks" (Faculty J)		
12 PM - "Op-ed Writing" (Faculty B)	12 PM - Noon conference	12 PM - Resident DEI committee meeting	12 PM - Noon conference	12 PM - Noon conference	
1 PM - "Intro to Practical Peds" (Faculty C&D)	1 PM - Smart Goals workshop	1 PM - NICU curriculum development (Faculty G)	1 -3 PM - Combined Fellows Conference- "Adult Learning Science"	1 PM - Scholarly activity and project time	
2 PM - "Intro to Elective and Milestones and Evaluation" (Faculty E)	2 PM - Journal club article selection/review	2 PM - Curriculum development (Faculty H)			
	3 PM - PP Project preparation	3 PM - Kern's textbook review	3 PM - Faculty Development Feed - choose your own podcast		
5:30 PM - "Academic Games and Teaching Technology"		4 PM - "Understanding by Design" (Faculty I)			
Monday	Tuesday	Wednesday	Thursday	Friday	
8:30 AM - Morning report	8:30 AM - Morning report	8:30 AM - Morning report	8:30 AM - Morning report	8 AM - Grand rounds	
9 AM - Resident Teaching Session - "Parent Phone Calls"	9 AM - Resident Teaching Session - "Procedure Skills" for Practical Peds	9 AM - Med Ed Leadership Panel	9 AM - "Mentoring" (Faculty M)	9-12 PM - CV update, choosing your track mentor, evaluation of the elective, Med ed track planning session	
		10 AM - "Clinical Decision Making" (Faculty L)	10 AM - "Closing Ceremonies"		
		11:15 AM - "Team Feedback" (Chiefs)			
11AM - Resident panel					
12 PM - QI noon conference	12 PM - Peer Assisted Learning Program (PALP) meet	12 PM - Noon conference	12 PM - M&M	12 PM - Noon conference	
1 PM - Coaching (and "Creating a Program that Works") Workshop! (Faculty E)	1 PM - Resident Teaching Session - "Teaching during Rounds"	1 PM - Self-study - "SMALL TEACHING" faculty development modules	1 PM - "Lifelong Learning Across the Continuum" (Faculty N)	1 PM - Resident teaching session - "NICU SIM"	
			3:30 PM - "Innovations in Med Ed" (Faculty O)		

**Table 6:** Medical Education Didactic Session Outline

Faculty-Led Didactic Session Topics	
Op Ed Writing	Team Feedback
Residency Milestones and Evaluation	Scholarly Frameworks
Academic Games/Teaching Tech	Adult Learning Science
Coaching Workshop	Mentoring
Teaching Mastery of Procedures	Lifelong Learning
Smart Goals Workshop	Innovations in Med Ed
Curriculum Development	Bloom’s Taxonomy and
Kern's Review	Securing your Future
Understanding by Design Med Ed Leadership Panel	Question Aid for Rich, Realtime Discussion
Resident-Led Sessions	
“Sick or not sick”	
Mock parent phone calls	
Teaching during rounds	
Handoffs	
Neonatal simulations	
Professional Development Workshops	
Journal club	
CV workshop	
Mentorship selection	

**Table 7:** Discussion Guide for Residents

Questions for Individual Sessions:
Did this session met the goals and objectives of the elective?
How did the session meet the goals and objectives of the elective?
Would you keep the didactics from this session for next year’s elective?
What, if anything, would you change/add to this day’s didactics for next year?
Please include any additional comments

**Table 8:** Discussion Guide for Faculty

Faculty Self-Evaluation
What is your name?
What role(s) do you perform in medical education in our department?
What was the title/topic of your session for the Medical Education Elective?
What were the objectives of your talk?
What is the ideal target audience for your talk?
Did your session reach the goals and objectives of the elective?
Based on your self-assessment of your session, what, if anything, would you change for the next rotation? Consider the overall goals of the rotation.
Are there any medical education topics/sessions that you think we should add or change for the elective?
